# Virtual residency recruitment: future directions in the new era

**DOI:** 10.1080/07853890.2022.2148732

**Published:** 2022-11-21

**Authors:** Daniel Breitkopf, Cheryll Albold, Jonathan Barlow, Venkatesh Bellamkonda, Claire Dorcent, Annie Sadosty, Michelle Clarke, Daniel Cabrera

**Affiliations:** Mayo Clinic School of Graduate Medical Education, Rochester, MN, USA

**Keywords:** Virtual interview, in-person interview, hybrid interview, recent pandemic, resident recruitment, resident selection, web conferencing platforms, emerging conferencing tools

## Abstract

**Introduction:**

The COVID-19 pandemic led to many changes in healthcare including graduate medical education (GME). Residency and fellowship programs halted in-person recruitment and pivoted to virtual models. Residency selection and recruitment were practices ripe for redesign, as they relied on in-person interviewing as the major point of contact prior to match list creation. In this commentary, we review the state of virtual interviewing and propose a future state where virtual interactions are commonplace and integrated into a comprehensive recruitment process.

**Discussion:**

Virtual recruitment has led to a reduction of expenses, improved time efficiency for all parties and a reduced carbon footprint. Residency match outcomes have not changed substantially with the advent of virtual interviewing. Hybrid approaches, including virtual and in-person options have significant drawbacks and pitfalls which may limit adoption. Given the upheaval in GME recruitment caused by the pandemic, and the limitations of current methods for candidate assessment and interactions with programs, further innovation is needed to achieve an optimal state for all stakeholders. Multiple technology innovations are on the horizon which may improve the ability to interact virtually. Adoption of new technology along with expanding the timeline for residency recruitment may further optimize the process for both applicants and programs.

**Conclusions:**

The GME community was able to adopt technology for the recruitment interview rapidly due to the pandemic. As more opportunities for technology-based interactions grow, the opportunity exists to reimagine recruitment beyond the interview. While resources are constrained, some of the efficiencies gained by adopting virtual interviewing can be leveraged to expand the interactions between programs and applicants. Incorporation of in-person interaction may still be needed. Models will need to be developed to build upon the best characteristics of the virtual and in-person environments to optimize GME recruitment.

**KEY MESSAGES:**
- Virtual communication methods have substantially changed residency recruitment during the COVID -19 pandemic.
- COVID -19 related changes in residency recruitment, including wide adoption of virtual methods, should be maintained and strengthened.
- Efforts should be made to advance the gains in residency recruitment strategy during the pandemic by use of technologies that expand virtual interactions beyond the interview.

## Introduction

The novel coronavirus 2019 (COVID-19) served as a catalyst for many changes in healthcare. Graduate medical education (GME) was no exception, as programs halted in-person recruitment and pivoted to virtual models. Residency selection and recruitment are practices ripe for redesign. For years, education leaders have discussed the challenges associated with the interview process such as effectiveness, expense, time away from learning, inequities, and bias while struggling to make meaningful changes [1]. Upon the declaration of the COVID-19 pandemic by the World Health Organization in March 2020, the GME community was compelled to change the residency recruitment model.

The Coalition for Physician Accountability (COPA) recommended virtual interviews to reduce the risks of travel during the COVID-19 pandemic [2]. Simultaneously, away rotations for medical students were suspended, eliminating an important method for students to audition for residency positions. Data from the 2021 National Residency Program Match (NRMP) program director survey indicated that 96% of interviews were conducted virtually [3]. Overall applicant match and program fill rates for PGY-1 slots in the 2021 and 2022 matches were not substantially changed compared to prior years [4,5]. Beyond the match and fill rates, concerns exist regarding appropriate candidate selection for an individual program in the virtual recruitment era. Longer term data will be needed to fully assess the impact of virtual recruitment on attrition and program completion. Nonetheless, as the pandemic continued, US national medical education organizations again recommended virtual interviews for the 2022 and 2023 residency match cycles [6,7].

Two years later, virtual recruitment and interviews are commonplace and prospective residents have adjusted. Innovation in residency recruitment has extended beyond the interview to include smartphone apps containing program information, virtual social events and social media to connect applicants with the program [8–10]. Still, considerable uncertainty and trepidation remain among programs on how to proceed in the future: should programs return to the traditional model, or should virtual interviews become the new standard? The goal of this commentary is to review the current state of virtual recruitment and re-imagine the future of GME recruitment using emerging technologies for virtual interactions.

## Discussion

Options for interviewing candidates include in-person interviews, virtual interviews using real time voice and video, and a hybrid combining virtual and in-person methods. Before COVID-19, most GME programs used in-person interviews. Travel bans and concerns for putting interviewees and faculty at risk of infection led to changes toward complete virtual methods. As the pandemic wanes, questions remain as to the value of the virtual versus in-person interview and the potential value of a hybrid approach.

### In-person interviewing

In-person interviews are characterized by the presence of both parties’ physical presence for the interaction. Surveys in several different specialties indicate that applicants and program directors prefer in-person interviews [11–15]. In addition to preferences for in-person interviews, concerns exist about the implications of virtual interviews on match outcomes. Virtual interviews resulted in a higher rate of matching at a student’s home institution [16,17]. There is evidence that some program directors are concerned that applicants from less prestigious schools are at a disadvantage in the era of virtual interviews and limited away rotations [12]. Both issues may have a substantial negative influence on diversity, equity, and inclusion, and the critical blending of cultures, both nationally and globally, through the match. Current virtual recruitment models may also hamper connecting applicants with the environment and true culture of training programs.

### Virtual interviewing

Virtual interviews involve synchronous interaction of the two parties *via* electronic means, usually consisting of real time video and audio feeds [18]. The most common platforms in use currently for virtual interviews are Zoom (Zoom Video Communications, Inc.; San Jose, CA), Skype (Microsoft Inc, Redmond, WA), Webex (Cisco Systems, Inc., San Jose, CA), and Facetime (Apple, Inc., Cupertino, CA). Advocates for virtual recruitment processes argue that this approach is applicant-centric allowing substantial cost savings, in addition to avoiding unnecessary travel and time away from medical school educational opportunities, as these two factors created the largest burden for the system [19,20]. Concurrent suspension of away rotations may have reduced barriers for economically disadvantaged applicants and applicants like osteopathic and international medical graduates who have historically not had equal access to these opportunities [21]. In-person interview attendance costs to the applicant over the season can easily exceed $10,000 [22]. In contrast, the virtual model and lack of away rotations during COVID-19 resulted in $6311 average savings per applicant [23]. The carbon footprint of residency interviewing has been estimated to be largely reduced by virtual methods [24–26]. Program associated costs of interviewing may also be reduced by use of virtual methods. Costs avoided may include both reduction of time spent in coordination of in-person events as well as the actual costs of meals and physical space usage.

With the virtual process, applicants may be able to schedule and attend more interviews without the burden of interview expenses. The virtual process may also decrease applicants’ anxiety levels by interviewing in the comfort of their own personal spaces. Surveys show that most interviewees feel they could present themselves well and assess programs in virtual-only format [20,27].

Fears that virtual interviews preclude full candidate assessment or communication of program attributes may be unfounded. Studies of the virtual interview process found that programs perceived they could present themselves and their program and assess applicant candidacy virtually [20,28]. During the pandemic, many institutions increased their social media presence to enrich the presentation of their program [29,30]. Despite early unfamiliarity with digital platforms, they are increasingly recognized as effective and efficient means of communication and assessment for recruitment [29,30]. There has been a move towards more holistic review rather than simple reliance on test scores for decisions regarding interviews [1,31]. Implementation of virtual interviewing has not seemed to alter the trend towards using all data on the application to make decisions regarding interviews.

### Hybrid approach to interviewing

With increasing likelihood that residency recruitment will not return to pre-pandemic state, another model is for a virtual interview combined with an on-site experience as a hybrid approach. The on-site experience may be an additional interview used in the program’s decision on ranking, or as an opportunity for the applicant to explore the program’s physical environment without being evaluated. Although a hybrid approach can offer an opportunity to incorporate more customary interview practices, such as social events and campus and facilities tours; a hybrid approach unravels many of the virtual recruitment benefits such as cost-effectiveness, reduced applicant and program expenses, and increased geographic applicant diversity. Furthermore, it presents at least two major risks and pitfalls.

First, post interview interactions and communications between applicants and programs increase opportunities for misinterpretation of commitment and therefore, risk breach of the National Residency Matching Program (NRMP) Match Participation Agreement governing such practices [32]. A hybrid approach, even with an optional on-campus or in-person component, risks inherent bias towards participants over non-participants. To avoid a match violation while deploying a hybrid model, universally accepted mechanisms would need to be in place limiting or prohibiting post-interview invitations for campus visits until after program rank order list submission deadlines have passed.

Second, there are risks of increasing inequity in a hybrid approach. Applicants may feel pressure to travel for increased visibility and perceptions of a campus visit influencing final ranking. There are concerns that some underrepresented minority (URM) applicants may feel a disproportionate obligation to travel versus their non-URM peers [7]. The NRMP could alter the due date for rank list submission, so that programs submit before applicants, allowing for in-person visits to the program without influence on a program’s rank list.

### Comparison of methods

A comparison of the three interview methods, focusing on the practical aspects, technological benefits and limitations is presented in Table 1. A comprehensive review of GME candidate assessment is beyond the scope of this commentary. Many of the studies comparing in-person interviews to virtual interviews were performed with older technologies. A meta-analysis found that interviewer ratings of candidates are lower overall with video interviews when compared to in-person [33]. The mechanism for a lower rating on video interviews may be due to several factors, such as differences in perceived social presence, eye contact and impression management [34]. These data highlight the risks of a dual approach to interviewing, as well as the need for standardization and improved virtual technologies for interviewing GME candidates.

**Table 1.** Comparison of interviewing methods.

	Advantages		Disadvantages	
Method	Applicant	Program	Applicant	Program
In-person	Evaluate physical space/location Social interactions facilitated Exposure to local culture/ethos	Concentration of resources in a smaller pool of interviewees	Travel cost Travel time Focusing on a smaller pool of programs	Difficulty accessing URM and other disadvantaged candidates* Operational expenses Negative impact of travel on environment
Virtual	Time efficiency Cost savings Interview at more programs	Opportunity to access URM and other disadvantaged candidates* Environmental impact lessened	Limit on evaluation of physical space/location Less exposure to culture/ethos	Increased application volume Learn new skill sets for virtual environment
Hybrid	Ability to access and screen programs Ability to focus on program of highest interest after virtual interview	Ability to clarify application information and identify better candidates Further evaluate candidates of highest interest after virtual interview	Travel time Travel cost Potential bias against candidates who chose to interview remotely	Potential NRMP violations Potential negative impact on recruiting disadvantaged candidates

*Groups included are underrepresented minorities in medicine (URM) and those who are economically or geographically disadvantaged, and international medical graduates.

NRMP: National Residency Match Program.

### Future technology adoption

Given the upheaval in GME recruitment caused by the pandemic, and the limitations of current methods for candidate assessment and interactions with programs, further innovation is needed to achieve an optimal state for all stakeholders. In our view, future GME recruitment should include a change in paradigm from an antiquated process, based on brief, in-person, and choreographed personal interactions, to a new system that is continuous, frequent, organic, and mostly based on digital interactions. This aligns with major industry-wide changes [35] and harmonizes with society-wide changes, including technology adoptions efforts to increase equity, diversity and inclusion, as well as decreasing market asymmetries during decision-making [36].

Looking to the horizon of the human connection landscape needed for virtual recruitment, creativity, connectivity, and technology are coming together in unique ways. Novel models build upon digital platforms allowing more natural and unintrusive mingling to facilitate spontaneous conversations: essentially virtual networking between people. These platforms allow applicants to learn about programs or interact with program leaders frictionlessly. The currently used technologies for virtual interviewing such as Facetime, Zoom, Skype and Webex all work to establish a direct 2-dimensional synchronous communication between people. In addition, digital collaboration tools like Slack (Slack Technologies, Inc, San Francisco, CA) and Teams (Microsoft Inc, Redmond, WA) add on options for asynchronous communication through text or chat features that exist even when the participants are not interacting at the same time. These platforms are commonly used for interviews and open houses; however, the execution can be unique. Mayo Clinic Emergency Medicine residents and candidates engage in interactive games and exercises through Zoom (personal communication, James Homme, MD). In addition, these applications can be used in other ways, for example, the Mayo Clinic Internal Medicine residency began using Zoom on a tablet device to allow approximately 900 applicants over the past two recruitment seasons to experience live clinical rounds to showcase their collegiality and patient focused culture (personal communication, Carrie Thompson, MD). In this way, novel uses for these newly adopted technologies may create more meaningful and natural connections between applicants and programs, providing applicants more insight into the overall culture of programs.

Building upon these platforms, there are tools that allow participants to move on a 2-dimensional space before interacting with each other. This allows virtual mingling and regrouping of people to facilitate spontaneous and variable conversations: essentially virtual networking between people. Remo (Remo, San Francisco, CA), founded in 2019, allows participants to move their avatar around a flat virtual set of tables and create different and spontaneous groupings of people who then have 2-dimensional video conferencing ability. Gather (Gather Presence, Inc., San Bruno, CA) takes a creative twist upon that and allows gamification of a similar concept. People can move avatars similar to video game characters around a large flat-world and create spontaneous interactions with others. The American Academy of Physical Medicine and Rehabilitation held a national recruitment event where multiple training institutions hosted virtual tables in a Remo environment and applicants were able to move from virtual table to table and learn about or interact with individual program leaders: just as they would have in an in-person residency fair event. Certainly, the effectiveness or impact of residency fair events is not formally proven, however, this example shows how technology is already being used to recreate traditional applicant-program encounters in virtual ways.

Evolving the experience from a synchronous 2-dimensional one to a 3-dimensional one, Cisco Systems Inc. is developing a version of Webex that permits users to see holographic versions of each other and interact with virtual 3D objects simultaneously. This technology is expected to be available for widespread use potentially in 2024 or even earlier. When the environment also becomes 3-dimensional, the metaverse may be more completely realized. Facebook (Meta, Menlo Park, CA) rebranded itself to Meta in 2021, taking a step into this future of metaverse where increasingly lifelike avatars of people interact with each other in virtual 3-dimensional worlds. Specific interweb platforms using hardware like Meta Quest (Meta, Menlo Park, CA), such as VRChat (VRChat Inc., San Francisco, CA) and RecRoom (Rec Room Inc., Seattle, WA), would permit training programs to create a virtual world or space and invite potential applicants to engage with them in that 3-dimensional multisensory environment (Table 2). With these new technologies, one could imagine having a virtual operating room that is identical to those that trainees can expect to learn within, showing them around, introducing them to a surgical team all in this metaverse. The current boom in technologic solutions for advancing human connection has been focused on only two senses: sight and hearing; however, there are likely coming solutions that use haptic devices to incorporate touch, smell, and possibly even taste into the future of human connection.

**Table 2.** Technologies for virtual recruitment interactions^+^.

Asynchronous	2D people	2D people/2D world	Holographic telepresence*	3D avatars/3D world
Slack	Zoom	Remo	Holographic Webex	VR Chat
Teams	Skype	Gather	Matsuko	Rec Room
Email	Facetime	Kumospace	DVE Holographics	Hubs
Text Messaging	Webex	Topia	Mesh	MeetinVR
	Teams	Branch		Horizon Workrooms
				

^+^This is not an exhaustive list of technologies, platforms, or companies. These are provided for example of the technology being referenced for illustrative purposes only.

*These technologies are in development and have not yet been brought to market.

These possibilities can surely feel distant, impossible, or impractical. An example of disruptive technology adoption is the smartphone, such as the Apple iPhone, introduced 15 years ago. This new technology, which integrated a portable music player, camera, cellular phone, and computer into a single device, in such a small portion of time, made email management, text messaging, social media interactions, remote learning all possible on a scale that was previously not present. We believe it is very possible that metaverse technology may also catalyze change in the way humans connect in the same or even shorter period.

### Reimagining residency recruitment in the new era

An effective residency match cannot occur with scant and extemporaneous data. A good relationship can only happen if it is based on deep knowledge built on rich and long interactions. The recruitment and matching process needs to become longitudinal, broad, and deep. The process may start earlier than the fourth year of medical school. Prospective applicants could regularly interact with residents, faculty, and program directors, attend didactics on-line, meet periodically with partners and families and learn about the community offerings. Expanded access to program leadership may require an incremental investment of time and effort. The Accreditation Council for Graduate Medical Education (ACGME) has recently redefined requirements for program director dedicated time; however, this additional time may not be sufficient to adopt the reforms we propose. Costs of acquiring modern technology may be a significant barrier to program adoption. Programs that expand their recruitment timeline should measure the effectiveness of their efforts and the incremental resources needed. Data from the early adopters of expanded recruitment practice will help to inform institutional leaders and ACGME as to the need for changes in faculty effort and other resources needed for success.

A longer relationship may provide improved data points. Medical students could have a better understanding of culture and operation of the program, grasp group dynamics, explore financial opportunities, and assess the community impact of each institution. For program directors, longer engagement may permit a global understanding of the applicant, including interactions with other learners and faculty, skill development and understanding the potential contributions of each applicant to the program.

## Conclusions

The future presents a bold opportunity to reimagine GME recruitment, seizing the opportunities created by the pandemic. New technologies may increase interactions beyond the interview and provide better data to programs and applicants. Adoption of new technology and processes will reduce some burdens on both applicants and programs, while requiring additional program resources. The role of in-person interactions in this new era is still unclear. Hybrid models may be adopted but with safeguards in place for the protection of all parties. Now is the time to build on the momentum created during a worldwide crisis and embrace a new virtual and forward-thinking recruitment model for GME programs.

## Data Availability

Data sharing is not applicable to this article as no new data were created or analyzed in this study.
